# Supplementary material for: Race, Adolescent Socioeconomic Status, and Lifetime Non-Medical Use of Prescription Painkillers: Evidence from the National Longitudinal Study of Adolescent to Adult Health
Source: Int J Environ Res Public Health. 2021 Nov 23;18(23):12289. doi: 10.3390/ijerph182312289 (PMC8657390; doi:10.3390/ijerph182312289)
Supplement: Supplementary file 1 [file ijerph-18-12289-s001.zip › ijerph-1466912-supplementary.pdf]

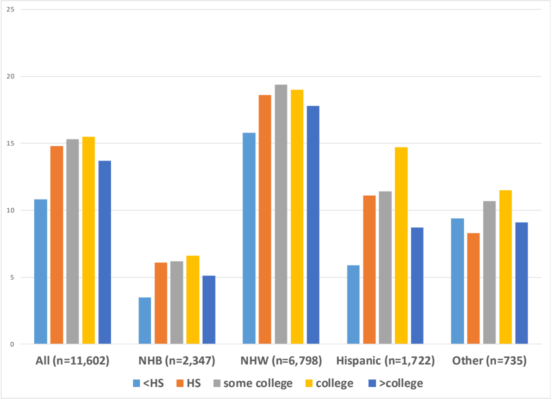

Figure S1. Prevalence of Reported NMUPP at Wave 4 by Parental Education Level (Adjusted for Age, Sex, Parental Income, and Region of US).

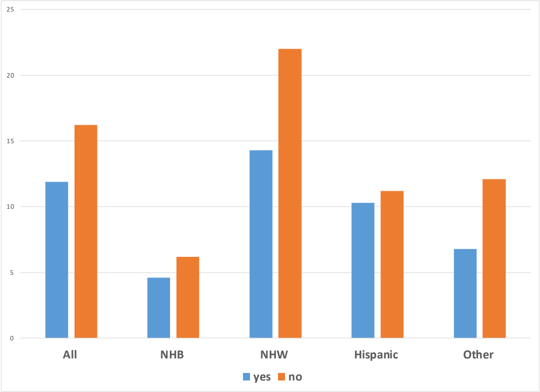

Figure S2. Prevalence of NMUPP by Home Ownership Status at Wave 4 (Adjusted for Age, Sex, Parental Income, Parental Education, and Region of US).

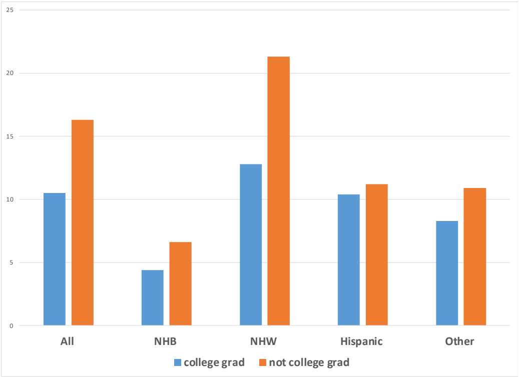

Figure S3. Prevalence of NMUPP by Own Education Level at Wave 4 (Adjusted for Age, Sex, Parental Income, Parental Education, and Region of US).

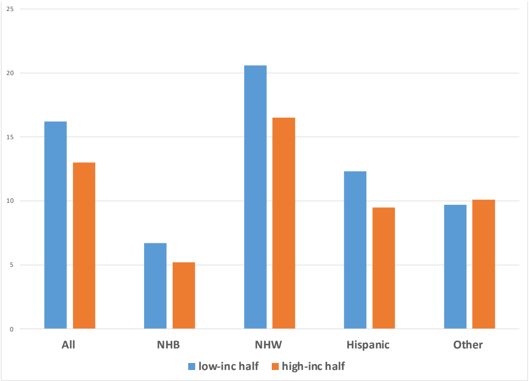

Figure S4. Prevalence of NMUPP by Own Pre-Tax Income at Wave 4 (Adjusted for Age, Sex, Parental Income, Parental Education, and Region of US).
